# Atractylenolide-I prevents abdominal aortic aneurysm formation through inhibiting inflammation

**DOI:** 10.3389/fimmu.2025.1486072

**Published:** 2025-01-31

**Authors:** Shuxiao Chen, Xiaotian Liu, Xincheng Zhou, Weixiao Lin, Minting Liu, Haoran Ma, Keli Zhong, Qiming Ma, Chengjian Qin

**Affiliations:** ^1^ Department of Bariatric Surgery, The First Affiliated Hospital of Jinan University, Guangzhou, China; ^2^ Clinical Medicine, International College of Jinan University, Guangzhou, China; ^3^ School of Stomatology, Jinan University, Guangzhou, China; ^4^ Department of Pathology, The First Affiliated Hospital of Jinan University, Guangzhou, China; ^5^ Department of Gastrointestinal Surgery, Shenzhen People’s Hospital (The Second Clinical Medical College, Jinan University; The First Affiliated Hospital, Southern University of Science and Technology), Shenzhen, Guangdong, China; ^6^ Department of General Surgery, The First Affiliated Hospital of Gannan Medical University, Ganzhou, China; ^7^ Department of Neurosurgery, Affiliated Hospital of Youjiang Medical University for Nationalities, Baise, China; ^8^ Key Laboratory of Medical Research Basic Guarantee for Immune-Related Diseases Research of Guangxi (Cultivation), Guangxi, China

**Keywords:** abdominal aortic aneurysm, atractylenolide-I, inflammation, AMPK, treatment

## Abstract

**Background:**

Abdominal aortic aneurysm (AAA) is a degenerative disease with high mortality. Chronic inflammation plays a vital role in the formation of AAA. Atractylenolide-I (ATL-I) is a major bioactive component of Rhizoma Atractylodis Macrocephalae that exerts anti-inflammatory effects in various diseases. The purpose of this study is to investigate the role of ATL-I in the progression of AAA.

**Methods:**

AAA was constructed in C57BL/6 mice by porcine pancreatic elastase (PPE)-incubation, and the diameter of the aorta was measured by ultrasound. ATL-I was administered by gavage on the second day after modeling to explore its significance in AAA. The pathological and molecular alteration was investigated by immunostaining, ELISA, qRT-PCR and Western blotting.

**Results:**

ATL-I inhibited the dilatation of the abdominal aorta and decreased the incidence of AAA. ATL-I alleviated the infiltration of macrophages in the adventitia and reduced the levels of proinflammatory factor IL-1β and IL-6 in the aorta and circulatory system, while increasing the expression of anti-inflammatory factor IL-10. Moreover, ATL-I restrained loss of smooth muscle cells and elastic fiber degradation by suppressing MMP-2 and MMP-9 expression. Mechanistically, phospho-AMPK expression was elevated in AAA groups, and ATL-I administration suppressed its expression to improve the pathological damage of aorta.

**Conclusions:**

ATL-I meliorated vascular inflammation by targeting AMPK signaling, ultimately inhibiting AAA formation, which provided an alternative agent for AAA treatment.

## 1 Introduction

Abdominal aortic aneurysm (AAA) is a severe vascular disease characterized by focal dilation of the abdominal aorta (1). Once AAA ruptures, the mortality rate is as high as 65-85% (2–4). With the development of open repair and endovascular aneurysm repair (EVAR) therapy, patients with diameters greater than 5.5 cm (women > 4.5 cm) can be effectively treated (5, 6). However, patients with small AAA or those deemed unfit for AAA repair criteria will gradually deteriorate. There is no successful pharmacological treatment to delay expansion or prevent rupture of AAA in humans (7). It highlights an emergent need to discover effective drugs to limit AAA growth.

In AAA development, the arterial wall suffers a destructive remodeling, including upregulation of matrix metalloproteinases (MMPs), loss of vascular smooth muscle cells (VSMCs), and degradation of elastic fibers (8). Chronic inflammation is closely related to vascular remodeling (9, 10). Infiltrated macrophages and VSMCs can secrete various proinflammatory cytokines, such as interleukin-1β (IL-1β) and IL-6, thereby activating more immune cells to aggravate the vascular injury, eventually resulting in probable rupture of AAA (11, 12).

Atractylenolide-I (ATL-I), a major bioactive component of Rhizoma Atractylodis Macrocephalae, exerts anti-oxidant, anti-inflammatory, and anti-tumor effects (13–15). Li et al. demonstrated that ATL-I had an intensely inhibitory effect on ox-LDL-induced inflammatory responses and a protective role in anti-atherosclerosis (16). Wang et al. observed that ATL-I inhibited the osteogenic differentiation of valve interstitial cells through blocking phosphatidylinositol-3-kinase (PI3K)/AKT pathway (17). Furthermore, Wang et al. discovered the promising therapeutic potential of ATL-I for sepsis-induced cardiomyopathy by suppressing macrophage polarization (18). Based on previous reports, we speculated that ATL-I might affect the formation of AAA by exerting anti-inflammatory effects.

In this study, we confirmed that ATL-I could alleviate the pathological lesion and lumen dilatation of AAA by targeting AMPK signaling, which provided a novel therapeutic strategy for patients with AAA.

## 2 Materials and methods

### 2.1 Animal experiments

Eight-week-old male C57BL/6J mice were obtained from the laboratory of Jinan University’s animal center and used in this study. All animals were fed in cages and bred in a temperature-controlled room maintained at 22 ± 1°C on a 12-h light-dark cycle with standard food and water. All animal experimental procedures complied with Institutional Animal Care and Use Committee of Jinan University.

### 2.2 Porcine Pancreatic elastase-induced AAA model

According to previously reported surgical methods (19), a mouse model of AAA or sham was induced by perivascular application of PPE (Sigma-Aldrich; 4 U/mg protein) or saline. Briefly, after induction of anesthesia with 3% isoflurane, the lower three-fourths of the infrarenal abdominal aorta was exposed and isolated from the vena cava. 30uL elastase was applied to exposed aortic adventitia and infiltrated for 30 minutes. Then, the abdominal cavity, especially the adventitia, needed to be rinsed with saline. Finally, the abdomen was closed routinely with 6-0 silk sutures. After 14 days, all mice were sacrificed by carbon dioxide inhalation, while the aortas were isolated for pathological examination.

### 2.3 Grouping and treatment of animals

**Figure 1:**
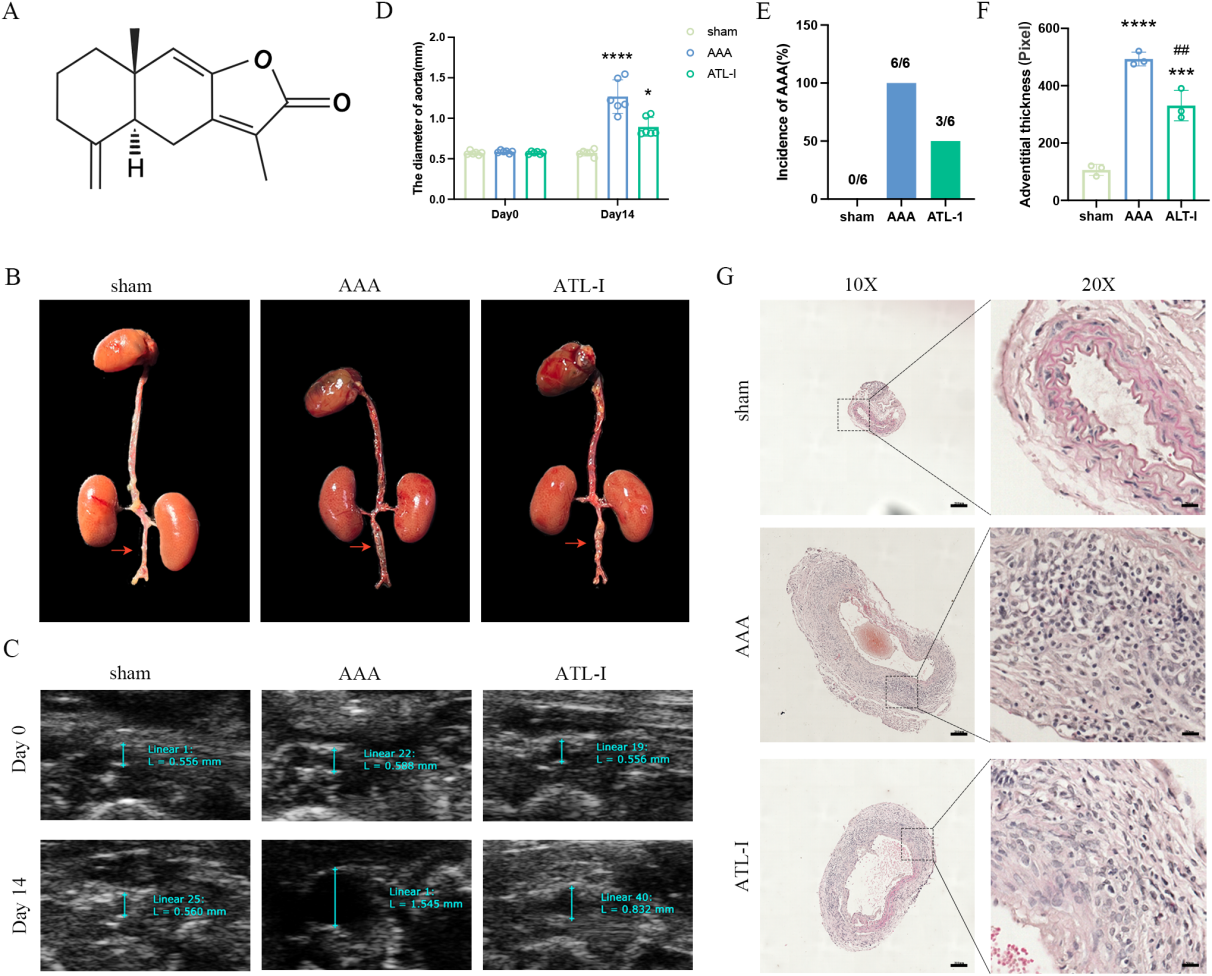
Effect of ATL-I on the abdominal aortic diameter. **(A)** Molecular structure of ATL-I. **(B)** Surgical anatomy diagram among the three groups. **(C)** Representative ultrasonography images of the three groups on the 0 and 14 days after modeling. **(D)** The diameter of abdominal aorta on the 0 and 14 days after modeling. **(E)** Incidence of AAA (represented by percentages). **(F, G)** Adventitial thickness of aorta and representative images of HE staining. *P < 0.05, ***P < 0.001, ****P < 0.0001 vs. the sham group; ^##^P < 0.01 vs. the AAA group.

To investigate the effect of ATL-I on AAA, a total of 18 mice were divided by random number table into sham group, AAA group, and ATL-I group with 6 samples in each group. After modeling, mice in ATL-I groups received the ATL-I (20mg/kg/day, 73069-13-3, MCE) by gavage daily for the next 14 days, and mice in AAA group were given the same amount of sterile PBS (20–23). The molecular structure of ATL-I was shown in **Figure 1A**.

To investigate whether ATL-I affects AAA through AMP-activated protein kinase (AMPK) signaling pathway, 24 mice were randomly divided into four groups of six mice in each group. The treatment of the sham group, AAA group and ATL-I group was the same as the groups mentioned above. In the ATL-I+CC group, mice were simultaneously given compound C (CC, 300mg/kg/day, 866405-64-3, MCE) and ATL-I (20mg/kg/day).

### 2.4 Aneurysm quantification

The diameter of the subrenal abdominal aorta was measured by B-ultrasound imaging (Vevo 2100, Visual Sonic Inc.) before and 14 days after modeling. After anesthesia with 1.5% isoflurane, the aorta of mouse was detected during systole by a blinded investigator. AAA was defined as the maximum diameter of the subrenal aorta that dilates more than 50% of its baseline (The diameter of the abdominal aorta examined before modeling) (24).

### 2.5 Histological analysis

Aortic samples isolated from the mice were fixed with 4% paraformaldehyde for 24h and embedded in paraffin. Serial sections (5μm each) were used for HE staining, elastic fiber staining (G1596, Solarbio) and immunofluorescence staining. The severity of elastic fiber degradation was evaluated on a standard described in a previous study: score 1, <25% degradation; score 2, 25% to 50% degradation; score 3, 50% to 75% degradation; score 4, >75% degradation (25).

### 2.6 Immunofluorescence staining

Paraffin slides were incubated in xylene and alcohol of different gradients for deparaffin and rehydration. The slides were placed in 10 mM sodium citrate buffer (pH 6, BL619A, Biosharp) at 85°C for 30 minutes and cooled at room temperature for 20 minutes. Then, the slides were incubated in 5% horse serum for 30 minutes at room temperature. Next, the slides were incubated at 4°C overnight with the following primary antibodies: CD68 (1:200, ab125212; Abcam), CD80 (1:200, ab254579, Abcam), CD206 (1:200, AF2535, R&D Systems), α-SMA (1:500, ab5694, Abcam). The following day, the slides were incubated with the corresponding secondary antibodies and DAPI at room temperature for 30 minutes. Immunofluorescence images were obtained using confocal laser scanning microscopy (Leica) and analyzed using Image-J software.

### 2.7 Enzyme-linked immunosorbent assay

Serum was obtained by centrifugation after blood collection through inferior vena cava. The content of IL-1β, IL-6, IL-10, alanine aminotransferase (ALT), aspertate aminotransferase (AST), creatine, and blood urea nitrogen (BUN) was analyzed using a commercially available ELISA kit (ml098416; ml098430; mIC50274-1; SP30122; CSB-E12649m; LE-M0613; RF8291; MLbio; Spbio; Cusabio; Isite; Ruifan) according to the manufacturer’s protocol. The results were calculated using the standard curve method and expressed as pg/ml.

### 2.8 Quantitative real-time polymerase chain reaction

Total RNA was extracted from the subrenal abdominal aorta using Trizol Reagent (155960-18, Ambion). Then, 1ug of total RNA was subjected to reverse transcription based on the manufacturer’s instructions. The mRNA expression of IL-1β, IL-6 and IL-10 in aorta was analyzed through PCR system (BIO-RAD). The primer sequences are shown in **Table 1**.

**Table 1:** Primer information table.

Gene name	Forward	Reverse
IL-6	CCGGAGAGGAGACTTCACAGA	AGAATTGCCATTGCACAACTCTT
IL-1β	GCAACTGTTCCTGAACTCAACT	ATCTTTTGGGGTCCGTCAACT
IL-10	GCTCTTACTGACTGGCATGAG	CGCAGCTCTAGGAGCATGTG
β-actin	GGCTGTATTCCCCTCCATCG	CCAGTTGGTAACAATGCCATGT

### 2.9 Western blotting

Total protein was extracted with RIPA lysate. Protein concentration was quantified by bicinchoninic acid (BCA) protein assay kit (P0010, Beyotime). The protein was separated by 10% SDS-PAGE, transferred to a polyvinylidene difluoride (PVDF) membrane, and incubated in 5% BSA for 1 hour at room temperature. Then, the membranes were incubated overnight with primary antibodies AMPK (1:1000, 2365, CST), p-AMPK (1:1000, 2535, CST), MMP-9 (1:1000, ab283575, Abcam), MMP-2 (1:1000, ab86607, Abcam), β-actin (1:1000, ab8226, Abcam) at 4°C. The membranes were incubated with secondary antibodies for 1 hour at room temperature the next day. Finally, the protein bands were visualized with the ECL system (BIO-RAD).

### 2.10 Statistical analysis

All data were presented as the mean ± SD and executed using GraphPad Prism 8.0 software. Statistical differences between groups were performed by one-way ANOVA. P<0.05 was considered as statistically significant.

## 3 Results

### 3.1 ATL-I inhibited PPE-induced AAA formation

To explore whether ATL-I could restrain AAA progression, we first detected the diameter of the infrarenal abdominal aorta on the 14^th^ day after modeling. As revealed by **Figure 1B**, the aorta diameter in the AAA group was significantly increased, which was reversed after ATL-I treatment. At the same time, ultrasound data showed that the infrarenal aorta was dilated with a maximum diameter of 1.23 ± 0.21 mm in the AAA group. However, the baseline of its diameter was 0.57 ± 0.02 mm, and the maximum infrarenal aortic diameter was only slightly increased to 0.91 ± 0.02 mm in the ATL-I group (**Figures 1C, D**). Ultimately, ATL-I decreased the incidence of AAA (**Figure 1E**). Additionally, we observed that ATL-I couldn’t change the levels of ALT, AST, creatine and BUN, indicating that it had no obviously toxic side effects on liver and renal function *in vivo* (**Supplementary Figures S1A–D**). HE staining results displayed that the adventitia was thicker, accompanied with recruitment of vast inflammatory cells in AAA, and the terrible condition was improved following ATL-I treatment (**Figures 1F, G**). Taken together, ATL-I administration inhibited the progression of AAA.

### 3.2 ATL-I suppressed inflammatory responses of AAA

**Figure 2:**
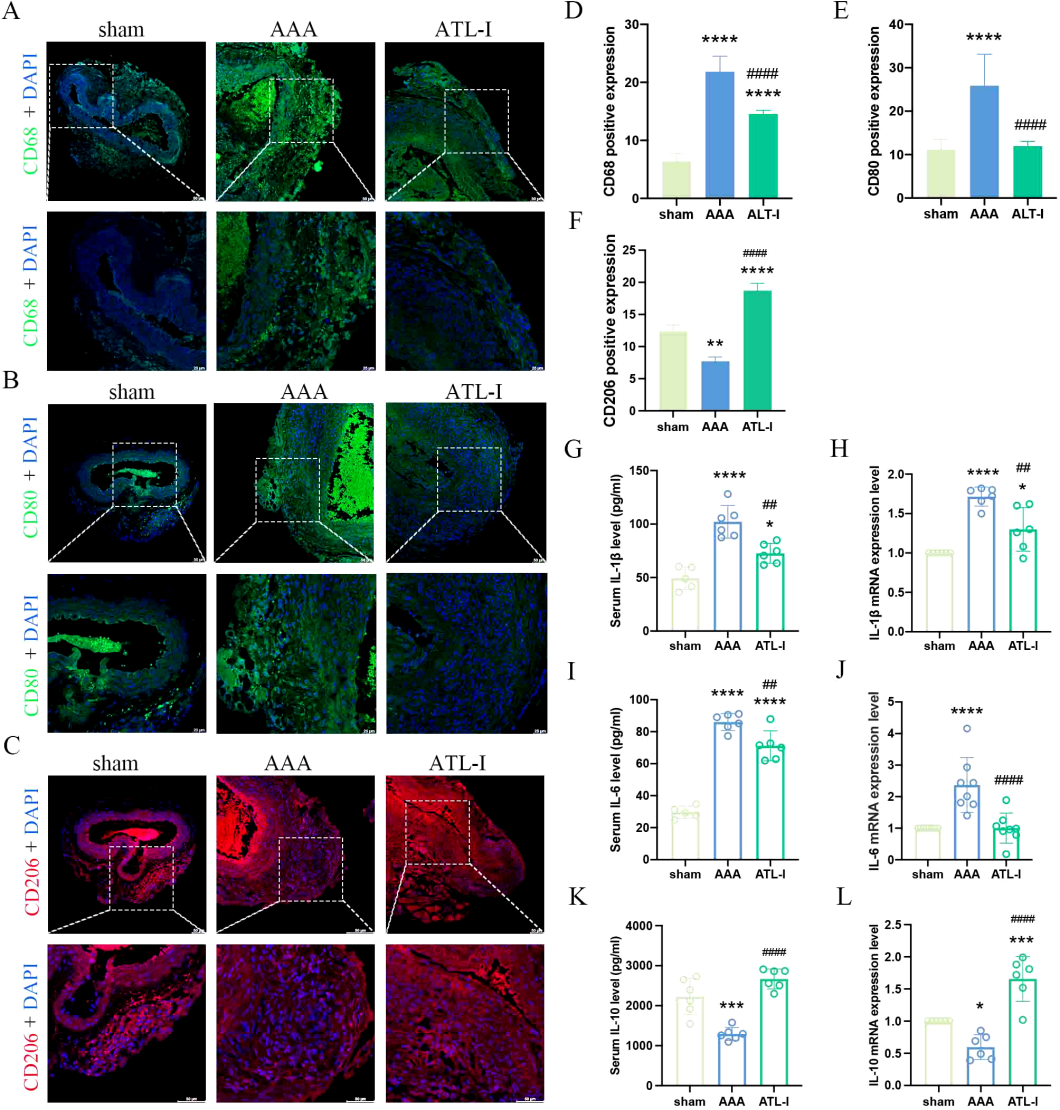
Effect of ATL-I on inflammatory responses in AAA. **(A)** Representative immunofluorescent images of CD68. **(B)** Representative immunofluorescent images of CD80. **(C)** Representative immunofluorescent images of CD206. **(D-F)** Densitometric analysis of CD68, CD80 and CD206. **(G)** Changes in circulating levels of IL-1β. **(H)** Relative mRNA expression of IL-1β in aorta. **(I)** Changes in circulating levels of IL-6. **(J)** Relative mRNA expression of IL-6 in aorta. **(K)** Changes in circulating levels of IL-10. **(L)** Relative mRNA expression of IL-10 in aorta. *P < 0.05, **P < 0.01, ***P < 0.001, ****P < 0.0001 vs. the sham group; ^##^P < 0.01, ^####^P < 0.0001 vs. the AAA group.

Inflammation has been considered as a central player in the progression of AAA. There were a large number of macrophages (CD68+) in the adventitia of AAA (**Figures 2A, D**). To examine whether the inhibitory effects of ATL-I on AAA progression were associated with changes in M1 or M2 macrophages, we subjected abdominal aortic tissue for immunostaining using CD80 to stain M1 macrophages and CD206 to identify M2 macrophages. As shown in **Figures 2B, E** the significantly increased CD8 staining was observed in the adventitial region of the aneurysm, which was markedly reduced following ATL-I administration. Nevertheless, ATL-I treatment significantly augment aggregation of M2 macrophages in adventitia (**Figures 2C, F**). Subsequently, we compared the differences in the levels of inflammatory cytokines among three groups using ELISA and qPCR experiments. As illustrated in **Figures 2G–J**, the expression of proinflammatory cytokines, such as IL-1β and IL-6, was elevated in the aneurysm and circulating system of mice with AAA, which was restrained after ATL-I treatment. By comparison, decreased anti-inflammatory cytokine IL-10 expression in AAA were reversed by ATL-I (**Figures 2K, L**). As such, our results demonstrated the anti-inflammatory activity of ATL-I on AAA.

### 3.3 ATL-I attenuated the damage of the aorta by inhibiting MMP-2 and MMP-9

**Figure 3:**
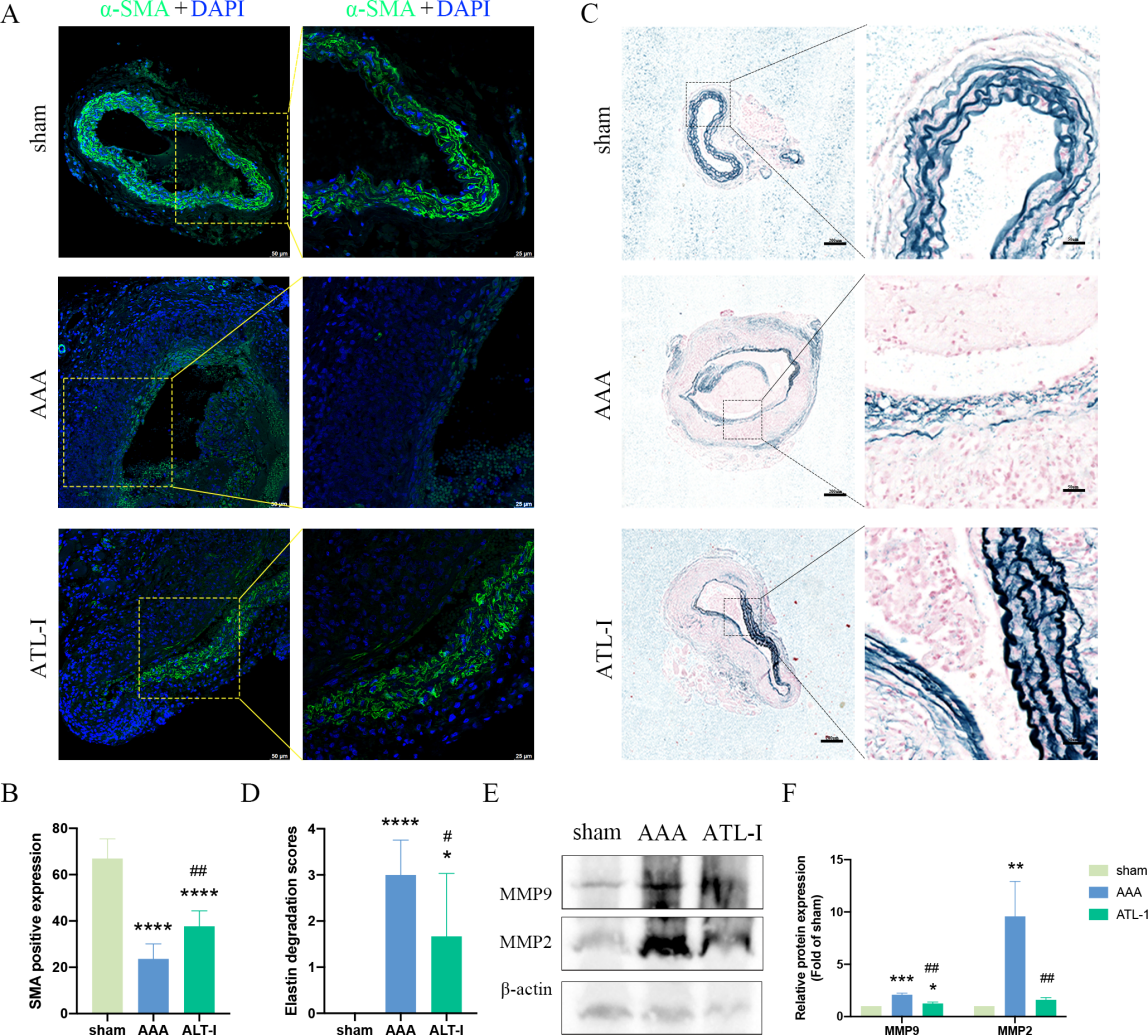
Effect of ATL-I on the histopathological lesion of the aortic wall. **(A)** Representative images of immunofluorescence. **(B)** Densitometric analysis of α-SMA. **(C)** Representative images of elastin staining. **(D)** Elastin degradation score. **(E, F)** Representative blots and semiquantitative analysis of MMP-2 and MMP-9. *P < 0.05, **P < 0.01, ***P < 0.001, ****P < 0.0001 vs. the sham group; ^#^P< 0.05, ^##^P< 0.01 vs. the AAA group.

Macrophages and VSMCs generate MMP-2 and MMP-9, therefore triggering steady destruction of extracellular matrix (ECM). We next examined α-SMA expression and elastin alteration. As shown in **Figures 3A, B**, a large amount of VSMCs was lost and remaining VSMCs were disordered in aneurysm, whereas the VSMCs disruption was markedly ameliorated after ATL-I supplement. Similarly, ATL-I administration improved the condition of elastin that showed obvious fracture and degradation in AAA tissue (**Figures 3C, D**). We further verified the levels of MMP-2 and MMP-9 using western blotting. MMP-2 and MMP-9 were elevated in the AAA group, which was mitigated after ATL-I therapy (**Figures 3E, F**). Collectively, these results suggested that ATL-I inhibited MMP-2 and MMP-9 expression, thereby attenuating pathological lesions of aorta.

### 3.4 ATL-I retarded the progression of AAA by targeting AMPK

**Figure 4:**
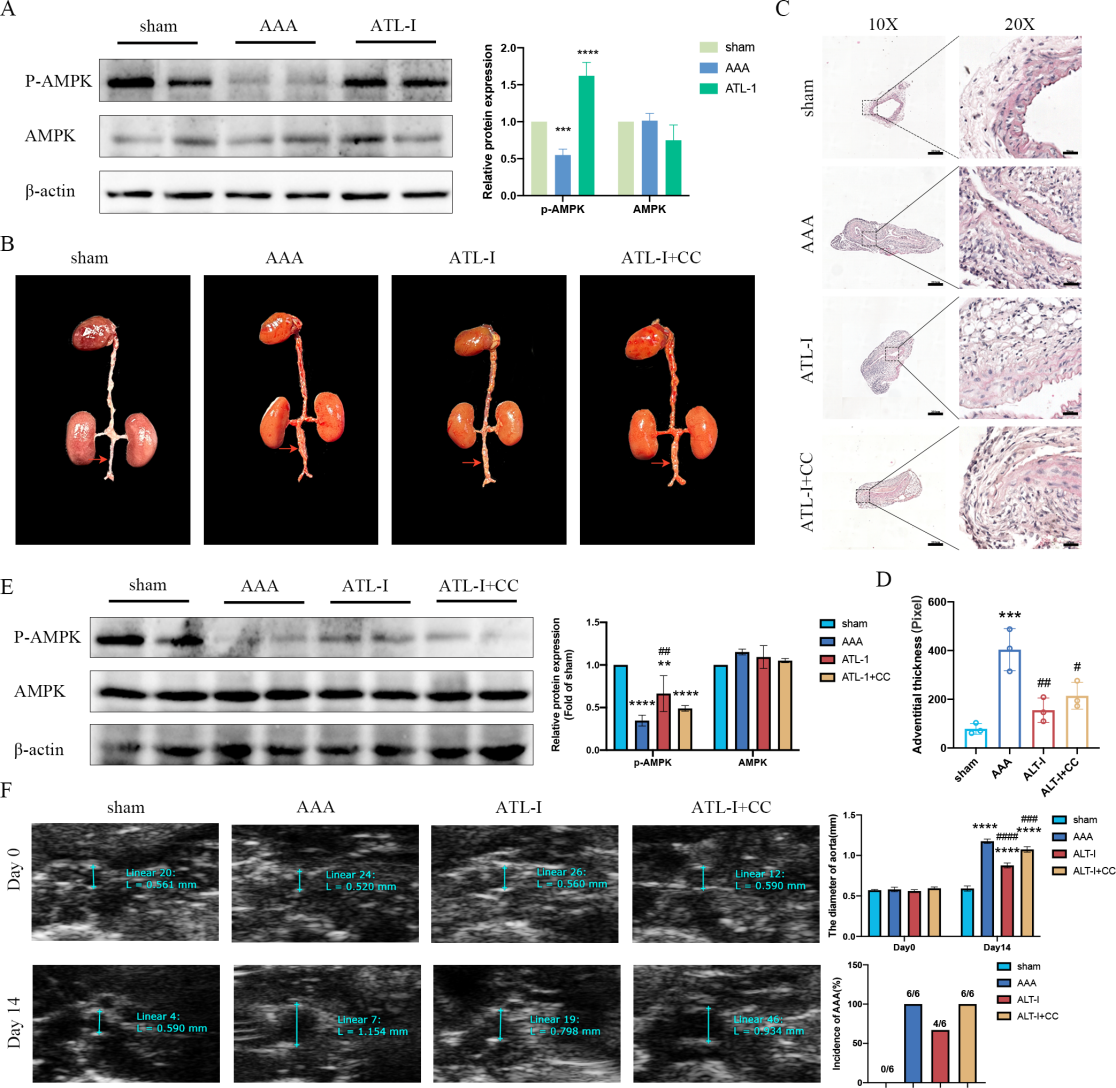
Effect of ATL-I on restraining the progression of AAA through AMPK pathway. **(A)** Representative blots and semiquantitative analysis of p-AMPK and AMPK. **(B)** Surgical anatomy diagram. **(C)** Representative images of HE staining. **(D)** Adventitial thickness of aorta. **(E)** Representative blots and semiquantitative analysis of p-AMPK and AMPK. **(F)** Representative ultrasonography images, the diameter of abdominal aorta, and the incidence of AAA. **P < 0.01, ***P < 0.001, ****P< 0.0001vs. the sham group; ^##^P < 0.01, ^###^P < 0.001, ^####^P < 0.0001 vs. the AAA group.

Though there was no difference in AMPK expression in three groups, the expression of phospho- AMPK (p-AMPK) was downregulated in AAA tissues, which was upregulated following ATL-I treatment (**Figure 4A**). Therefore, we speculated that ATL-I might exert its inhibitory effect on AAA by activating AMPK phosphorylation. As expected, the administration of compound C, an inhibitor of AMPK, abolished the salutary effects of ATL-I on AAA. It was found that the diameter of aneurysm and adventitial thickness were apparently increased in the ATL-I+CC group (**Figures 4B–D**). Compound C reduced the phosphorylation level of AMPK, and led to a larger diameter of abdominal aorta and a higher incidence of AAA, compared with ATL-I group (**Figures 4E, F**). Thus, ATL-I treatment retarded AAA formation partially by targeting AMPK.

### 3.5 ATL-I alleviated the inflammatory response via activating AMPK signaling

**Figure 5:**
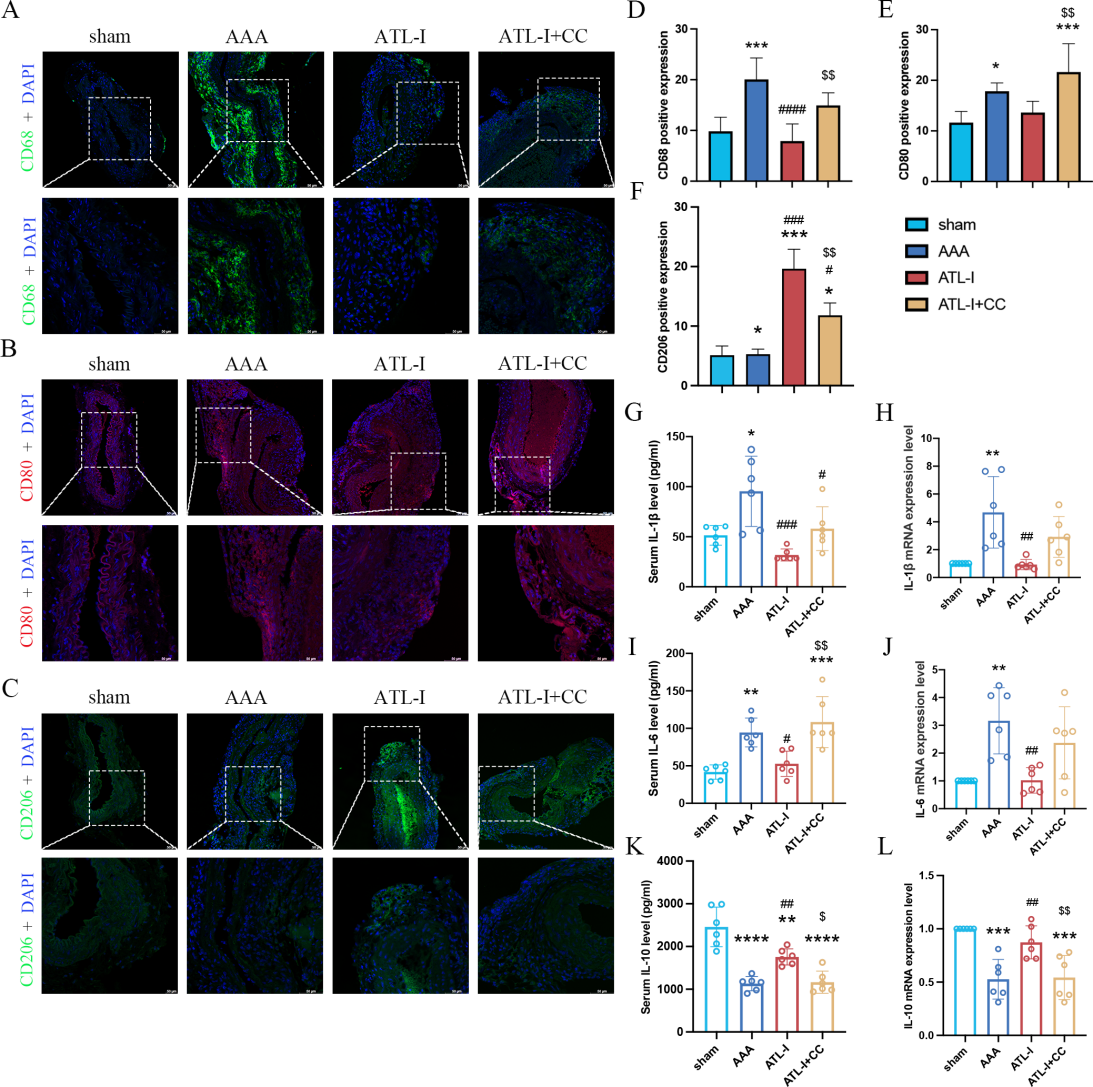
Effect of ATL-I on inhibiting inflammatory response of AAA through AMPK pathway. **(A)** Representative immunofluorescent images of CD68. **(B)** Representative immunofluorescent images of CD80. **(C)** Representative immunofluorescent images of CD206. **(D-F)** Densitometric analysis of CD68, CD80 and CD206. **(G)** Changes in circulating levels of IL-1β. **(H)** Relative mRNA expression of IL-1β in aorta. **(I)** Changes in circulating levels of IL-6. **(J)** Relative mRNA expression of IL-6 in aorta. **(K)** Changes in circulating levels of IL-10. **(L)** Relative mRNA expression of IL-10 in aorta. *P < 0.05, **P < 0.01, ***P < 0.001, ****P < 0.0001 vs. the sham group; ^#^P < 0.05, ^##^P < 0.01, ^###^P < 0.001, ^####^P < 0.0001 vs. the AAA group; ^$^P < 0.05, ^$$^P < 0.01 vs. the ATL-I group.

Subsequently, we examined the levels of macrophages and inflammatory cytokines after the application of compound C. Compared with the ATL-I group, the fluorescence intensity of CD68 was robustly increased in ATL-I+CC group (**Figures 5A, D**). Similarly, compound C reduced the inhibitory effect of ATL-I on M1 macrophages (**Figures 5B, E**). In contrast, increased CD206 expression was observed in the adventitia in the ATL-I group, which was restricted by compound C administration (**Figures 5C, F**). Meanwhile, ATL-I-induced decreased levels of IL-1β and IL-6 in the aorta and circulating system were reraised after the administration of compound C (**Figures 5G–J**). Our data also demonstrated that ATL-I-triggered the activation of IL-10 was significantly decreased following compound C treatment (**Figures 5K, L**). Taken together, our results supported the notion that AMPK was required for ATL-I to exert its anti-inflammatory effects on AAA.

### 3.6 ATL-I reduced the pathological injury by activating AMPK signaling pathway

**Figure 6:**
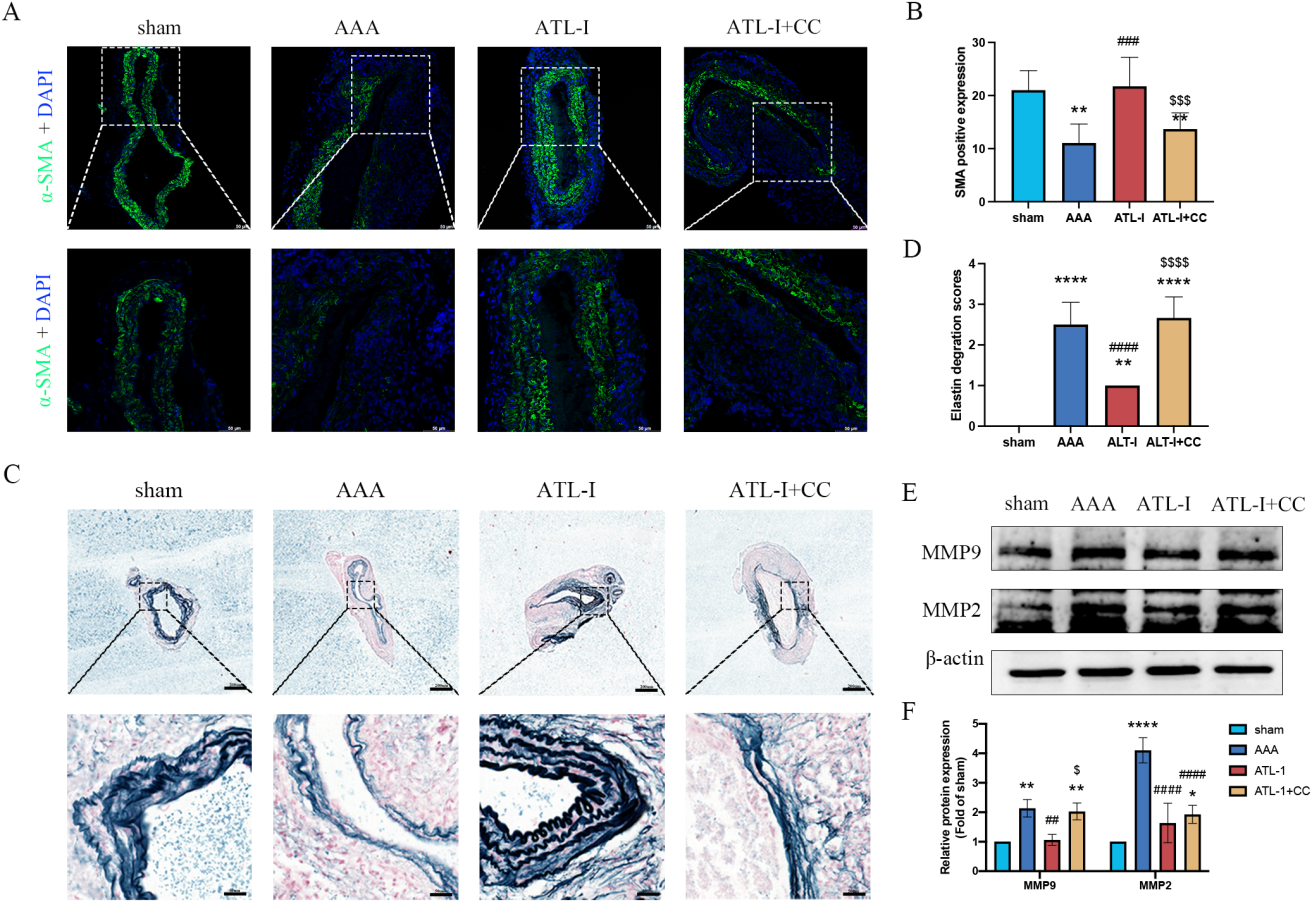
Effect of ATL-I on suppressing histopathological lesion of AAA through AMPK pathway. **(A)** Representative images of immunofluorescence. **(B)** Densitometric analysis of α-SMA. **(C)** Representative images of elastin staining. **(D)** Elastin degradation score. **(E, F)** Representative blots and semiquantitative analysis of MMP-2 and MMP-9. *P < 0.05, **P < 0.01, ****P < 0.0001 vs. the sham group; ^###^P< 0.001, ^####^P < 0.0001 vs. the AAA group; ^$^P < 0.05, ^$$$^P< 0.001, ^$$$$^P< 0.0001 vs. the ATL-I group.

Using immunostaining and elastic fiber staining, we observed that the α-SMA expression was dampened in the ATL-I+CC group, accompanied by the aggravated deterioration of elastic fibers, which was in line with pathological injury results of AAA as described above (**Figures 6A–D**). Furthermore, we noticed that the supplement of compound C impaired the inhibitory effects of ATL-I on MMP-2 and MMP-9 expression, as displayed in **Figures 6E, F**. Collectively, these findings suggested that ATL-I mitigated the pathological lesion of AAA by activating AMPK signaling pathway.

## 4 Discussion

AAA is a severe disease affecting human health. With the improvement of living standards and the aggravation of the aging population, the incidence of AAA has increased yearly and is up to 4-8% in elderly people over 50 years old (26, 27). The development of AAA is very insidious, and the early patients have no apparent symptoms (28). Once the aneurysm is ruptured, patients present with severe abdominal pain, only 50% of which have the opportunity for emergency treatment. Even so, the 30-day mortality rate is as high as 30%-70% (29, 30). The clinical treatment and management of AAA remains quite a considerable challenge. Therefore, it is of great significance for early diagnosis and intervention of these patients.

Currently, drug therapy using natural substances has received increasing attention (31). Traditional Chinese medicines (TCMs) are considered a promising future for disease therapy by virtue of minimal side effects, low costs, and high efficiency. Prior studies have found that ATL-I, a biologically active herb known as Baizhu, exerts protective effects in a variety of diseases (32–34). ATL-I ameliorated cancer cachexia through decreased the phosphorylation levels of STAT3 and PKM2 (33). ATL-I acted as an inhibitor of TLR4, NF-κB, and MAPK signaling pathways, alleviating acetaminophen-induced acute liver injury (35). Additionally, ATL-I elevated the contents of neurotransmitters (5-HT, dopamine, and norepinephrine) through binding to 5-HT2A, thus improving depression-like phenotypes (36). However, there is no study on the ATL-I on AAA progression.

The pathogenesis of AAA is usually relevant to inflammation and ECM remodeling. A waterfall-like inflammation cascade ascribes to secretion of proinflammatory factors, which recruit more macrophages and lymphocytes to the adventitia (10, 37). Macrophages, VSMCs and fibroblasts secrete large amounts of MMP-9 and MMP-2 to aggravate ECM breakdown (11, 38). Many TCMs have been proven to inhibit the pathological progression of AAA. Paeonol, a peony bark extract, prevented experimental AAA progression by inhibiting the NF-κB pathway (39). Betanin exerted anti-inflammatory activity and ROS scavenging ability in AAA progression (40). At present, many experiments *in vivo* and *in vitro* have demonstrated the remarkable anti-inflammation efficacy of ATL-I, and revealed that its mechanism of action was associated with inhibition of inflammatory cells and medium. It was reported that ATL-I dose-dependently inhibited a variety of inflammatory mediator, such as nitric oxide (NO), tumor necrosis factor-α (TNF-α), IL-1β, IL-6, vascular endothelial growth factor (VEGF), and placental growth factor (PlGF) (41–43). Li et al. showed that ATL-I inhibited the proliferation and migration of VSMCs induced by oxidized low density lipoprotein (ox-LDL), and simultaneously reduced the production of mononuclear chemotactic protein 1 (MCP-1) to ameliorate the development of atherosclerotic (16). We thought that ATL-I had anti-AAA potential. As anticipated, ATL-I reduced the degeneration of elastic fibers in the aorta caused by PPE, and inhibit the lumen dilatation, thereby curbing AAA formation. Further researches confirmed that the infiltration of M1 macrophages was reduced, and the M2 differentiation of macrophage was increased following ATL-I administration. At the same time, the levels of pro-inflammatory factors (IL-1β and IL-6) decreased, and the expression of anti-inflammatory factor IL-10 upregulated. More importantly, ATL-I decreased the secretion of MMP-2 and MMP-9, thereby weakening the ECM degeneration. Whether ATL-I has inhibitory effect on lymphocytes deserves further investigation. Collectively, the protective effect of ATL-I on AAA was attributed partially to its anti-inflammatory activity.

AMPK is a serine/threonine kinase in which α subunits (α1 and α2 subtypes) have catalytic activity, while β and γ subunits are involved in maintaining the stability of the heterotrimer complex (44). AMPK, as an energy sensor, plays a role in regulating intracellular energy balance. AMPK is also responsible for influencing the biological process of autophagy, cell proliferation and ECM anabolism (45–47). However, AMPK dysfunction exists in a variety of diseases, including obesity, diabetes, and cardiovascular diseases. Studies have shown that the expression of p-AMPK is decreased in AAA, which contributes to the elevation of MMP-2/9 (48, 49). Mammalian target of rapamycin (mTOR), as a downstream molecule of AMPK, was elevated in AAA, but an inhibitor of mTOR was demonstrated to restrain the progression of AAA. Given the crucial role of AMPK in the biological activity of VSMCs, AMPK might act as a promising intervention target for the prevention and treatment of AAA. In this study, ATL-I could activate AMPK signaling and down-regulated MMP-2/9 levels to inhibit AAA progression, which was rescued after supplementing with AMPK inhibitor. Some studies have shown that JAK2/STAT3 and TLR4 are molecular targets of ATL-I and are also key molecules in AAA progression (35, 50–54). Therefore, when exploring the mechanism of ATL-I regulation of AAA, we try to find other molecular targets to improve the innovation of this study. In subsequent studies, we will continue to explore whether ATL-I also inhibits AAA progression through JAK2/STAT3 or TLR4 pathway in AAA, so as to further clarify the molecular mechanism of ATL-I on AAA. Although ATL-I interrupts the inflammatory cascade, the origin of the initial inflammation in the AAA needs to be further investigated. Moreover, developing ATL-I based on Chinese herbal extracts or active ingredients for clinical application is another strategy worthy of attention.

## Data Availability

The datasets presented in this study can be found in online repositories. The names of the repository/repositories and accession number(s) can be found in the article/**Supplementary Material**.

## Supplementary material

Supplementary Figure S1
**(A)** Serum ALT(U/L). **(B)** Serum AST(U/L). **(C)** Serum creatine (umol/L). **(D)** Serum BUN (mmol/L).
