# Supplementary figures and images for: Atractylenolide-I prevents abdominal aortic aneurysm formation through inhibiting inflammation
Source: Front Immunol. 2025 Jan 31;16:1486072. doi: 10.3389/fimmu.2025.1486072 (PMC11825332; doi:10.3389/fimmu.2025.1486072)

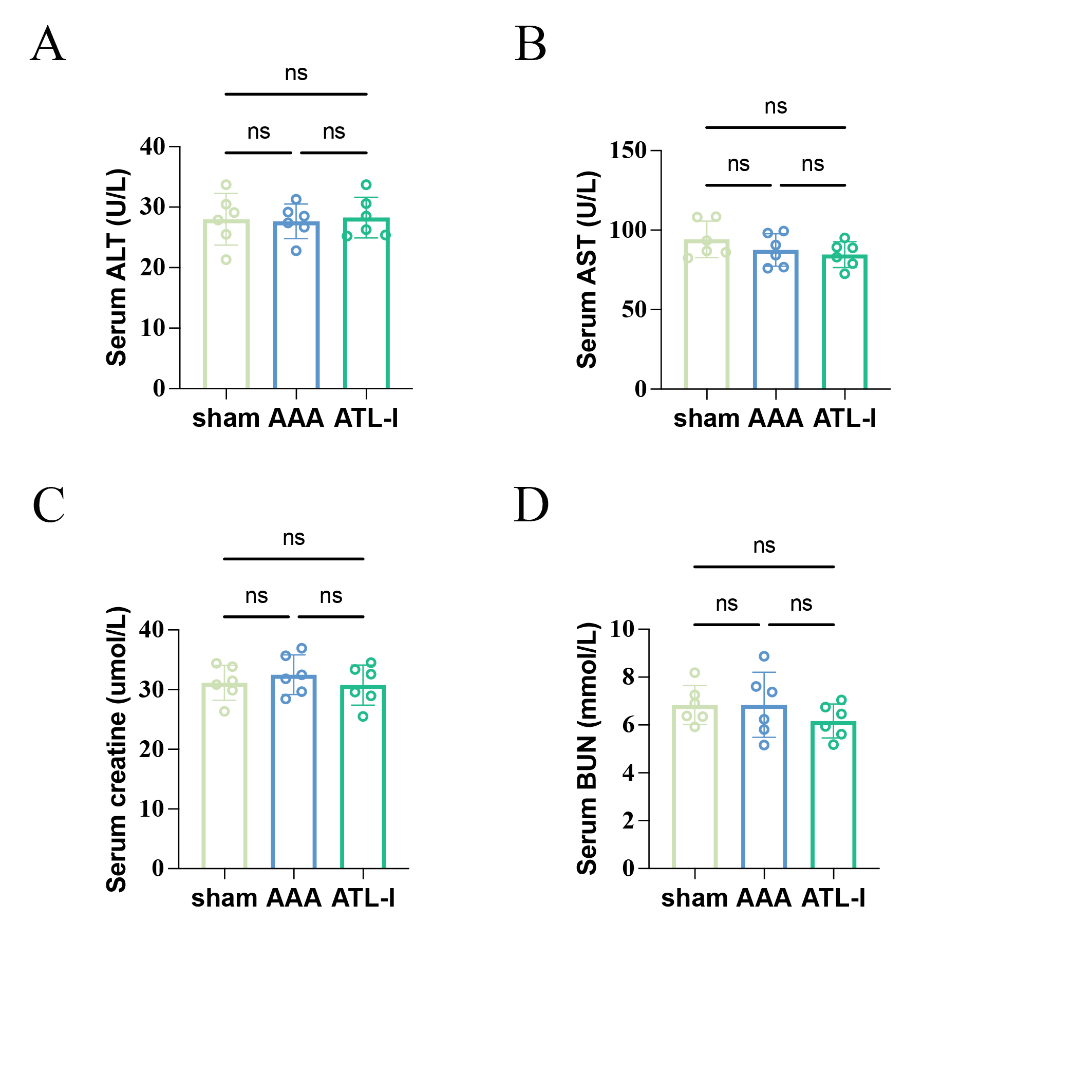

Supplement: Supplementary Figure S1 — (A) Serum ALT(U/L). (B) Serum AST(U/L). (C) Serum creatine (umol/L). (D) Serum BUN (mmol/L). [file Image1.tif]
